# Hydrogen Sulfide Ameliorates Angiotensin II-Induced Atrial Fibrosis Progression to Atrial Fibrillation Through Inhibition of the Warburg Effect and Endoplasmic Reticulum Stress

**DOI:** 10.3389/fphar.2021.690371

**Published:** 2021-12-07

**Authors:** Heng-Jing Hu, Xiu-Heng Wang, Yao Liu, Tian-Qing Zhang, Zheng-Rong Chen, Chi Zhang, Zhi-Han Tang, Shun-Lin Qu, Hui-Fang Tang, Zhi-Sheng Jiang

**Affiliations:** ^1^ Department of Cardiology Laboratory, First Affiliated Hospital of University of South China, Hengyang, China; ^2^ Postdoctoral Research Station of Basic Medicine, University of South China, Hengyang, China; ^3^ Department of Nuclear Medicine Lab, First Affiliated Hospital of University of South China, Hengyang, China; ^4^ Institute of Cardiovascular Disease and Key Lab for Arteriosclerology of Hunan Province, University of South China, Hengyang, China

**Keywords:** atrial fibrosis, atrial fibrillation, angiotensin II, endoplasmic reticulum stress, hydrogen sulfide, warburg effect

## Abstract

Atrial fibrosis is the basis for the occurrence and development of atrial fibrillation (AF) and is closely related to the Warburg effect, endoplasmic reticulum stress (ERS) and mitochondrion dysfunctions-induced cardiomyocyte apoptosis. Hydrogen sulfide (H_2_S) is a gaseous signalling molecule with cardioprotective, anti-myocardial fibrosis and improved energy metabolism effects. Nevertheless, the specific mechanism by which H_2_S improves the progression of atrial fibrosis to AF remains unclear. A case-control study of patients with and without AF was designed to assess changes in H_2_S, the Warburg effect, and ERS in AF. The results showed that AF can significantly reduce cystathionine-*γ*-lyase (CSE) and 3-mercaptopyruvate thiotransferase (3-MST) expression and the H_2_S level, induce cystathionine-*β*-synthase (CBS) expression; increase the Warburg effect, ERS and atrial fibrosis; and promote left atrial dysfunction. In addition, AngII-treated SD rats had an increased Warburg effect and ERS levels and enhanced atrial fibrosis progression to AF compared to wild-type SD rats, and these conditions were reversed by sodium hydrosulfide (NaHS), dichloroacetic acid (DCA) or 4-phenylbutyric acid (4-PBA) supplementation. Finally, low CSE levels in AngII-induced HL-1 cells were concentration- and time-dependent and associated with mitochondrial dysfunction, apoptosis, the Warburg effect and ERS, and these effects were reversed by NaHS, DCA or 4-PBA supplementation. Our research indicates that H_2_S can regulate the AngII-induced Warburg effect and ERS and might be a potential therapeutic drug to inhibit atrial fibrosis progression to AF.

## Introduction

Atrial fibrillation (AF) is the most common malignant cardiac arrhythmia, affecting 2% of the general population worldwide (Xiong et al., 2019), especially those over 70 years old (Piatek et al., 2016). It is expected that more than 20 million people worldwide will be affected by AF by 2050 (Christophersen et al., 2017). AF is a major risk factor for cardiovascular events, stroke and sudden death, imposing heavy financial and health care burdens on both individuals and society. Atrial fibrosis is a hallmark feature of AF, promotes atrial structural remodelling, such as induced left atrial dysfunction (LADS), and atrial electrophysiological remodelling, such as increased susceptibility to AF. The AF caused by renin-angiotensin-aldosterone system (RAAS) is closely related to the progression of atrial fibrosis. RAAS inhibitors can inhibited the occurrence and development of AF induced by atrial fibrosis and realized significant benefits for the long-term survival of AF patients (Han et al., 2013; Turin et al., 2018). However, these conventional drugs cannot completely cure atrial fibrosis (McMurray et al., 2012). Therefore, we are currently working on studying the mechanisms and interventions of atrial fibrosis to reduce the atrial structural remodelling and electrical remodelling caused by atrial fibrosis and reduce the occurrence and development of AF.

The endoplasmic reticulum (ER) is a multifunctional signalling organelle with complex stress signalling pathways and regulates the transport and apoptosis of cell membrane proteins, thereby achieving the correct folding of proteins (Welchen and Gonzalez, 2016). Induced dysfunction of the ER under glucose deficiency, hypoxia, inflammation and oxidative stress, which interfere with protein folding, posttranslational modification and secretion, is called ER stress (ERS). PKR-like ER kinase (PERK/eIF2AK3), inositol-requiring enzyme 1 (IRE1) and activating transcription factor 6 (ATF6) are three transmembrane ER signalling proteins mediated by ERS and the unfolded protein response (UPR). When ERS occurs, increase PERK, IRE1 and ATF6 protein expression and activity (Xin et al., 2011; Yan et al., 2019). Subsequently, phosphorylated PERK (p-PERK) can cause eukaryotic initiation factor 2*α* (eIF2*α*) phosphorylation (p-eIF2*α*) and increase activating transcription factor 4 (ATF4) expression, thereby inhibiting mRNA translation and global protein synthesis and promoting apoptosis (Wen et al., 2017). In experimental models and patients with AF, protein production and breakdown of dysfunctional proteins is disrupted, which contributes to myocardial remodelling and AF susceptibility (Deshmukh et al., 2015; Wiersma et al., 2017; Yuan et al., 2020). Furthermore, recent studies have demonstrated that local uncommon energy metabolism, such as insulin resistance and impaired glucose transport and uptake, leads to the accumulation of unfolded proteins in the lumen of the ER, which can lead to ERS (Luoma, 2013; Sun et al., 2019). The Warburg effect refers to a state in which tissues produce energy through glycolysis despite the presence of normal aerobic conditions, and increased local lactate accumulation is characteristic of the Warburg effect. An acidic environment can be induced in cardiovascular diseases (CHDs) and myocardial fibrosis and is closely related to ERS (Cottrill and Chan, 2013; Imle et al., 2019).

Current research has defined hydrogen sulfide (H_2_S), a toxic gas with the smell of rotten eggs, as the third gas signalling molecule after nitric oxide (NO) and carbon monoxide (CO). Cystathionine-*γ*-lyase (CSE), cystathionine-*β*-synthase (CBS) and 3-mercaptopyruvate thiotransferase (3-MST) are the three most common ways to synthesize hydrogen sulfide. CBS is a key enzyme for H_2_S production in the cardiovascular system (Leffler et al., 2006), and the CSE/H_2_S pathway plays an important role in regulating CHD (Pan et al., 2012). In addition, 3-MST is a key enzyme for H_2_S production in mitochondria (Beltowski, 2015). As a gaseous signalling molecule, H_2_S participates in maintenance of the physiology of the cardiovascular system through its anti-oxidant, anti-inflammatory, neuromodulatory, energy metabolism regulation (Liang et al., 2015), and cardioprotective. Therefore, it can improve atrial remodelling and AF (Xue et al., 2020) and myocardial ischaemia-reperfusion injury (Snijder et al., 2013) and has been shown to promote myocardial repair and long-term cardiac function recovery after infarction in CSE-KO mice (Ellmers et al., 2020). In addition, attenuation of pathological fibrosis by modulating signals involved in cardiac fibrosis is an area of great interest due to its obvious therapeutic implications for the treatment of AF (Su et al., 2021). It is well established that reduced H_2_S levels are associated with cardiomyocyte apoptosis, myocardial fibrosis, and ERS induced by ischaemia-reperfusion injury (Snijder et al., 2013; George et al., 2017; Ren et al., 2019). Nevertheless, to the best of our knowledge, it is not clear whether H_2_S can attenuate AngII-induced Warburg effect and ERS, thereby ameliorating atrial fibrosis progression to AF.

In this study, we hypothesized that H_2_S exerts cardioprotective against AngII-induced Warburg effect and ERS and further affects atrial fibrosis progression to AF. The mechanism was investigated using patient tissue specimens, rat models of AF, and HL-1 cell models of AF, with the aim of providing new molecular targets for prevention and treatment of AF.

## Materials and Methods

### Human Left Atrial Appendage

Human research was approved by the ethics committee of the First Affiliated Hospital of University of South China and was performed in compliance with the Declaration of Helsinki. Left atrial appendage (LAA) tissue specimens were collected from rheumatic heart disease (RHD) patients undergoing mitral regurgitation surgery in the First Affiliated Hospital of University of South China. Informed consent for clinical observation experiments was signed by the patient or a family member before surgery. Patients with benign/malignant tumours, acute/chronic heart failure, hypertension, chronic obstructive pulmonary disease, pulmonary heart disease, CHD, cardiomyopathy, hyperthyroidism and obesity as well as overweight patients and patients with diabetes mellitus (including type 1 and type 2 diabetes mellitus and impaired glucose tolerance) were excluded before enrolment. According to the presence or absence of AF, the enrolled patients were divided into a permanent AF group (*n* = 6) and a sinus rhythm (SR) group (*n* = 6). General patient information is shown in Table 1.

**TABLE 1:** Main characteristics of 12 patients with rheumatic heart disease with mitral valve replacement combined with AF or SR.

Parameter	AF patients	SR patients	P
Number of patients	6	6	
Sex (male/female)			
male	3	3	
female	3	3	
Age, y	58.1 ± 12.9	57.9 ± 13.2	0.62
Systolic blood pressure, mm Hg	121 ± 14	122 ± 13	0.91
Diastolic blood pressure, mm Hg	76 ± 12	75 ± 10	0.96
Body mass index, kg/m2	24.5 ± 3.4	24.3 ± 3.0	0.58

### Animal Models

In female animals, the incidence of supraventricular tachycardia is closely related to menstrual cycle, more common in luteal phase, and negatively related to estrogen level (Gowd and Thompson, 2012). Therefore, in order to exclude the effect of estrogen, combined with relevant AF research reports (Chan et al., 2019; Ge et al., 2020), we select male Sprague–Dawley (SD) rats, weighing 200–250 g, purchased from the Animal Department of University of South China, as research target. The animal studies were approved by the Ethics Committee of the First Affiliated Hospital of University of South China. Ang-II (200 ng/kg/min) was subcutaneously infused into the rats using a mini-pump (Model 2004; Alzet) for 4 weeks to induce atrial fibrosis (Ge et al., 2020). AF was induced by transesophageal stimulation. The inducibility of AF was clarified by atrial rapid programmed stimulation (ARPS) via oesophageal electrodes after 4 weeks of continuous Ang-II administration with a subcutaneous mini-pump as previously described (Song et al., 2019). AF vulnerability was assessed by stimulation for 5 s per cycle starting at 50 ms of the RR interval and ending at 2 ms decrements until termination at 10 ms decrements and resting for 5 s if no AF occurred. If AF occurred, the time from the end of stimulation to the first sinus P recovery, which was defined as AF duration, was recorded. To recover the sinus P wave, another stimulation of the next perimeter was performed until stimulation ended at 10 ms. AF was defined as a rapid and irregular atrial rhythm with irregular RR intervals lasting at least 1 s on ECG. AF was considered inducible if one or more bursts in the series caused an AF event. The number of AF episodes and AF duration were recorded and analysed. Minipump implantation and ARPS stimulation with oesophageal pacing were performed under 2% v/v isoflurane/oxygen inhalation anaesthesia. When ARPS stimulation was finished and the heart rate returned to the SR P wave, the SD rats were euthanized using potassium chloride (10% concentration, 75–150 mg/kg KCl administered via rapid intravenous injection) until cardiac function stopped. The left atrial tissue was stored in liquid nitrogen.

### Animal Experimental Design

SD rats were randomly divided into eight groups with six rats in each group. SD rats in each group received the following treatments (see Supplementary Figure S1).

Sham group: SD rats were infusion of 0.9% saline (200 ng/kg per minute) using continuous subcutaneous mini-pump for 4 weeks, and then ARPS stimulation was given through the oesophagus.

Ang-II group: SD rats were infusion of Ang-II (200 ng/kg per minute) using continuous subcutaneous mini-pump for 4 weeks, and then ARPS stimulation was given through the oesophagus (Ge et al., 2020).

Ang-II + NaHS group: SD rats were infusion of Ang-II (200 ng/kg per minute) using continuous subcutaneous mini-pump combined with intraperitoneal injection of NaHS (56 μM/kg qd) for 4 weeks, and then ARPS stimulation was given through the oesophagus (Pan et al., 2014).

Ang-II + 4-PBA group: SD rats were infusion of Ang-II (200 ng/kg per minute) using continuous subcutaneous mini-pump combined with intraperitoneal injection of 4-PBA (20 mg/kg qd) for 4 weeks, and then ARPS stimulation was given through the oesophagus (Luo et al., 2015).

Ang-II + DCA group: SD rats were infusion of Ang-II (200 ng/kg per minute) using continuous subcutaneous mini-pump combined with intraperitoneal injection of dichloroacetic acid (DCA, 50 mg/kg qd) for 4 weeks, and then ARPS stimulation was given through the oesophagus (Sun et al., 2016).

NaHS group: SD rats were infusion of NaHS (56 μM/kg qd) using continuous intraperitoneal injections for 4 weeks, and then ARPS stimulation was given through the oesophagus.

4-PBA group: SD rats were infusion of 4-PBA (20 mg/kg qd) using continuous intraperitoneal injections for 4 weeks, and then ARPS stimulation was given through the oesophagus.

DCA group: SD rats were infusion of DCA (50 mg/kg qd) using continuous intraperitoneal injections for 4 weeks, and then ARPS stimulation was given through the oesophagus.

### HL-1 Experimental Design

The HL-1 cell line was purchased from Shanghai (TongPai, China), used for *in vitro* research and cultivated in DMEM containing 10% foetal bovine serum (Gibco, MA, United States), 0.1 mM norepinephrine and 2 mM L-glutamine in a 37°C cell incubator with 5% CO_2_. Before each experiment, HL-1 cells were plated in six-well plates and treated as described below when the cells reached 70–80% confluence. (see Supplementary Figure S2):

**Control group:** HL-1 cells were cultured in DMEM for 24 h.

**Ang II group:** HL-1 cells were treated with Ang II (200 nM) for 24 h (Lee et al., 2020).

**Ang II + NaHS group:** HL-1 cells were pre-incubated with NaHS (100 μM) for 24 h, and then treated with Ang-II for another 24 h (Lee et al., 2019).

**Ang II + 4-PBA group:** HL-1 cells were pre-incubated with 4-PBA (500 μM) for 24 h, and then treated with Ang-II for another 24 h (Yuan et al., 2020).

**Ang II + DCA group:** HL-1 cells were pre-incubated with DCA (1.5 mM) for 24 h, and then treated with Ang-II for another 24 h (Li et al., 2019a).

**NaHS group:** HL-1 cells were pre-incubated with NaHS (100 μM) for 24 h, and then incubated with DMEM for another 24 h.

4-PBA group: HL-1 cells were pre-incubated with 4-PBA (500 μM) for 24 h, and then incubated with DMEM for another 24 h.

**DCA group:** HL-1 cells were pre-incubated with DCA (1.5 mM) for 24 h, and then incubated with DMEM for another 24 h.

### Cell Viability Assay

CCK-8 assays were used to investigate the viability of HL-1 cells cultured in 96-well plates. Our experimental methods were performed as described in a previous report by Li et al. (2020).

### Detection of Intracellular ROS

Intracellular ROS levels were determined by the oxidative conversion of cell-permeable DCFH-DA to fluorescent dichlorofluorescein (DCF). Our experimental methods were performed as previously reported by Thakur et al. (2015).

### Detection of Left Atrial Function

The patients and rats were placed on a test bench on the left side and connected with a synchronous 12-lead ECG. The left atrial diameter (LAD), left atrial diameter (LASED) and left ventricular ejection fraction (LVEF) were measured by the M-mode echocardiography. End systolic left atrial volume (LAESV) and end diastolic left atrial volume (LAEDV) were measured by the biplane Simpson’s method at apical four chamber and apical two chamber views. The left ventricular outflow tract velocity time integral (LVOT-VTI) was measured by the left ventricular outflow tract blood flow spectrum. The mitral annular motion spectrum (Ea, Aa) were measured by the tissue Doppler, and calculated the Ea/Aa ratio.

The body surface area (BSA) of patient’s was calculated according to the Stevenson’s formula (Si et al., 2018).

BSA = 0.0061 * height (CM) + 0.0128 * weight (kg) - 0.1529.

The body surface area of the rats was calculated using the Meeh-Rubner formula (Spiers and Candas, 1984).

BSA = K * weight 7) 2/3÷10,000, and K value in rats = 9.1.

The left atrial end-systolic volume index (LAESVI) was calculated as LAESVI = LAESV/BSA.

The left atrial ejection fraction (LAEF) was calculated as LAEF = (LAESV-LAEDV)/LAESV.

The left atrial function index (LAFI) was finally obtained as LAFI = (LAEF*LVOT-VTI)/LAESVI.

### Western Blotting

Collected the total protein of left atrium tissue and HL-1 cells in lysis buffer (Beyotime P0013F). Protein concentration was determined using a BCA protein assay kit (Beyotime P0012S). Equal amounts (70 μg) of protein were separated in a 12% sodium dodecyl sulfate (SDS)-polyacrylamide gel. Proteins were transferred to polyvinylidene difluoride (PVDF) membranes (Beyotime FFP33), blocked in Tris-buffered saline (TBS) with 5% milk/0.1% Tween 20 and incubated overnight at 4°C with anti-pyruvate dehydrogenase kinase 4 (PDK-4) (1:500, Abcam ab89295), anti-PDH (1:5,000, Abcam ab172617), anti-lactate dehydrogenase (LDHA) (1:1,500, Abcam ab222910), anti-matrix metallopeptidase 9 (MMP-9) (1:500, Abcam ab58803), anti-p-PERK (1:2000, Affinity DF7576), anti-PERK (1:1,000, Affinity AF5304), anti-p-eIF2α (1:1,000, Cell Signaling, #9721), anti-eIF2*α* (1:1,000, Cell Signaling, #9722), anti-Caspase-12 (1:1,000, Abcam ab8117), anti-ATF 4 (1:1,000, Abcam ab23760), anti-C/EBP homologous protein (CHOP, 1:200, Abcam ab11419), anti-CBS (1:800, Abnova H00000875-M01), anti-CSE (1:800, BIOSS bs-9515R), anti-3-MST (1:500, Abcam ab224043) or anti-glyceraldehyde 3-phosphate dehydrogenase (GAPDH, 1:5,000, Abcam ab 9,484) antibody in blocking buffer. The membranes were washed in 0.1% Tween/TBS and incubated with HRP-labelled goat anti-rabbit IgG (H + L) (Beyotime A0208) or HRP-labelled goat anti-mouse IgG (H + L) (Beyotime A0216) secondary antibody for 2 h at room temperature, followed by detection of chemiluminescence. Gel Imaging system (Bio-Rad Laboratories, Inc.) and Quantity One software (version 4.6.6; Bio-Rad Laboratories, Inc.) were used to image and analyse the western blot bands. Band intensities were detected with Super Signal West Pico Chemiluminescent Substrate (Thermo, United States).

### Myocardial Masson Staining

Fresh tissue was fixed in 4% paraformaldehyde for more than 24 h. The tissue was taken out of the fixative solution and placed in a fume hood for trimming of the target site tissue with a scalpel, and the trimmed tissue and the corresponding label was placed in a dehydration box. The dehydration box was put into a hanging basket, and the trimmed tissue was treated with a sequential alcohol gradient for dehydration in a dehydrator. Wax-soaked tissue was then embedded in an embedding machine. First, the melted wax was placed into the embedding frame, and before the wax solidified, the tissue was removed from the dehydration box and put into the embedding frame according to the requirements of the embedding surface, and the corresponding label was attached. The samples were cooled in a refrigerator at −20°. After the wax solidified, the wax block was removed from the embedding frame and trimmed. The trimmed wax block was sliced on a paraffin microtome to a thickness of 4 μm. The slices were floated on a spreader in warm water at 40°C to flatten the tissue, and the tissue was picked up with a glass slide and placed in a 60°C oven to bake for subsequent histochemical staining. The steps for Masson staining were as follows. Samples were fixed with neutral formalin, sectioned, deparaffinized in absolute alcohol, stained with the iron hematoxylin A liquid and B liquid (according to 1:1) for 3 min, differentiation for a few seconds with 1% hydrochloric acid alcohol, 60% ammonia water return blue and washed with water, stained with Ponceau red dye for 7 min and washed with distilled water slightly, differentiation for 3 min in Phosphomolybdate acid, stained with Aniline blue liquid for 5 min and differentiation for 1 min in glacial acetic acid solution, dehydrated in absolute alcohol, putting in xylene for transparency, and mounted with neutral gum. Collagen fibers were bluish in color and myocardial cell were orange. All sections with Masson staining were observed and the pictures were photograPEd by an Olympus microscope (IX-70 OLYMPUS, Japan) (Li et al., 2019b).

### Assessing the Levels of Lactic Acid, Glucose Consumption and Cellular ATP

After various treatments, 10 μl of homogenate (1:10 dilution) was collected and analysed using a lactic acid kit (NanJing Jiancheng Corp, A019-2), a glucose consumption kit (Sigma MAK083) and an ATP assay kit (Cloud Clone Corp, CEA349Ge) according to the manufacturers’ instructions (Xiao et al., 2017).

### Programmable Electrical Stimulator Detection of AERP and AERPd

Electrophysiological investigation was performed as previously described (Hu et al., 2019).

### Detection of HL-1 Cell Apoptosis *via* Flow Cytometry

The degree of apoptosis in HL-1 cells cultured in 6-well plates was detected by flow cytometry. Our experimental method was performed as previously reported by Gu et al. (Gu et al., 2018).

### Detection of Oxidative Damage by Assessing 8-OHdG Content in HL-1 Cell Mitochondrial DNA

Mitochondrial DNA oxidative damage was evaluated by detecting the 8-hydroxy-2-deoxyguanosine (8-OHdG) content. Our experimental method was performed as previously reported by Ni et al. (2021).

### Test of Carbonyl Protein Content in HL-1 Cells

After various treatments, HL-1 cells were collected and lysed, and the carbonyl protein content in HL-1 cells was measured as described by Ni et al. (2021).

### Determination of Lipid Peroxidation Level in HL-1 Cells

After various treatments, HL-1 cells were collected and lysed, and the lipid peroxidation levels in HL-1 cells were determined as previously reported by Ni et al. (2021).

### Determination of Mitochondrial Respiratory Function

The mitochondrial oxygen consumption in HL-1 cells was assayed based on the method described by Sun et al., 2018. Mitochondrial respiration buffer (125 mmol/L KCl, 5 mmol/L K2HPO4, 20 mmol/L MOPS, 2.5 mmol/L EGTA, 1 μmol/L Na4P2O7, and 0.1% bovine serum albumin, pH 7.4). Mitochondrial oxygen consumption was measured by Clark type oxygen electrode (Hansatech Instruments, Norfolk, United Kingdom) in mitochondrial respiration buffer at 30°C. Pyruvate (5 mmol/L) and malate (5 mmol/L) were used as substrates for complex I-containing mitochondria at a final concentration of 500 μg protein/ml. ADP-stimulated oxygen consumption (state three respiration) was measured in the presence of 200 μmol/L ADP, and ADP independent oxygen consumption (state four respiration) was also monitored. The respiratory control ratio (RCR, state three divided by state 4) reflects oxygen consumption by phosphorylation (coupling). The ADP/O ratio (number of ADP molecules added for each oxygen atom consumed) is an index of the efficiency of oxidative phosphorylation. State three shows the oxygen consumption of the ADP phosphorylation process during transformation to ATP. State four indicates ADP consumption after the base oxygen consumption and reflects invalid oxygen consumption.

### Quantitative PCR

Total RNA was isolated from cultured cells using TRIzol^®^ reagent (Tiangen Biotech Co., Ltd.). First-strand cDNA was synthesized from 4 mg total RNA using M-MLV reverse transcriptase (Promega Corporation) and oligo (dT). The cDNA for collagen I*α*, collagen III*α* and GAPDH was amplified using specific primers and conditions. The PCR products were subjected to electrophoresis in 1% agarose gels and visualized with ethylene bromide. Real-time quantitative PCR (qPCR) was performed with SYBRPremix Ex Taq (Perfect Real Time) (Takara, Japan). The relative mRNA levels were calculated by normalization to GAPDH, according to the 2-ΔΔCT method. The following specific primers were used: forward 5′-GTC​GTA​TCC​AGT​GCG​TGT​C-3′ and reverse 5′-GTG​GAG​TCG​GCA​ATT​GCA-3′; collagen IIIα, forward 5′-CAA​TTC​CTG​GCG​TTA​CCT​TG-3′ and reverse 5′-AGC​CCT​GTA​TTC​CGT​CTC​CT-3'; GAPDH, forward 5′-GGC AAGGTCATCCCAGAG CT-3′ and reverse 5′-CGCCTGCTT CACCACCTTCT-3'.

### Determination of the H_2_S Level in Patient and Rat Plasma

The plasma of patients and rats in each group was collected, and detection was performed according to the Methylene blue method described by Geng Bin et al. (Zheng et al., 2012).

### Measurement of CSE Activity

Prepare NaHS as standard curve: set the concentration gradient of NaHS standard to 1000, 800, 400, 200, 100, 50μM, and use deionized water instead of 0 μM. After configuration, add 500 μl each to the centrifuge tube, add 2.5 ml deionized water to each tube, then add 1% zinc acetate (400 μl), N,N-dimethyl-p-phenylenediamine sulfate (20 mM; 40 μl) in 7.2 mol/l HCl was added, which was immediately followed by the addition of FeCl3 (30 mM; 40 μl) in 1.2 mol/l HCl. Subsequently, the absorbance at 670 nm was measured using a microplate reader (Molecular Devices, LLC). The standard curve of NaHS is Y = 0.00038x + 0.09475, r2 = 0.99951. Following treatment, HL-1 cells were collected and homogenized in 50 mM ice-cold potassium phosphate buffer (pH, 6.8). Each 1 ml of the reaction mixture contained potassium phosphate buffer (100 mM; pH, 7.4), L-cysteine (10 mM), pyridoxal 5′-phosphate (2 mM) and cell lysis solution. In the central pool, 1% zinc acetate (400 μl) was added to trap the evolved H_2_S. The reaction was performed in tightly stoppered cryovial test tubes in a shaking water bath at 37°C. After incubating for 120 min, the zinc acetate was collected, and N,N-dimethyl-p-phenylenediamine sulfate (20 mM; 40 μl) in 7.2 mol/l HCl was added, which was immediately followed by the addition of FeCl3 (30 mM; 40 μl) in 1.2 mol/l HCl. Subsequently, the absorbance at 670 nm was measured using a microplate reader (Molecular Devices, LLC). According to the standard curve, the protein concentrations of Control and each treatment group were calculated, and the release of endogenous H_2_S was calculated. The amount of H_2_S present was standardized according to the protein concentration in the Control and each treatment groups. The experiment was repeated ≥6 times (Hu et al., 2021).

### Statistical Analyses

All the values are expressed as the mean ± standard error of the mean (SEM) or a percentage of at least three independent experiments. Statistical analysis was performed with unpaired Student’s t-test for comparisons between two groups. One-way analysis of variance (ANOVA) (Tukey’s) was used to evaluate AF duration, AERPd, AERP, ATP production, glucose consumption, lactate production, cell apoptosis, reactive oxygen species, carbonyl protein, 8-OHdG, respiratory control rate (RCR), ADP/O ratio, mitochondrial state3 oxygen consumption, mitochondrial state4 oxygen consumption and the expression of PDK-4, LDH, PDH p-PERK, ATF4, p-elf2*α*, CHOP, CSE caspase-12 and MMP-9. Fisher’s exact test was used to evaluate AF inducibility. *p* values <0.05 were considered statistically significant. All statistical analyses were performed using GraphPad Prism six software (GraphPad Software Inc., La Jolla, CA, version 6.42). The number of experiments per group is indicated in the figure legends.

## Results

### AF and SR Patient Characteristics

Table 1 shows the clinical characteristics of six patients with SR and six patients with AF. LAA were obtained from the 12 patients through cardiothoracic mitral valve replacement surgery (male, 50%, female, 50%). There were no significant differences in baseline data between SR and AF patients (Table 1)*.*

### Specific Changes in LAA Tissue in Patients With AF

**FIGURE 1:**
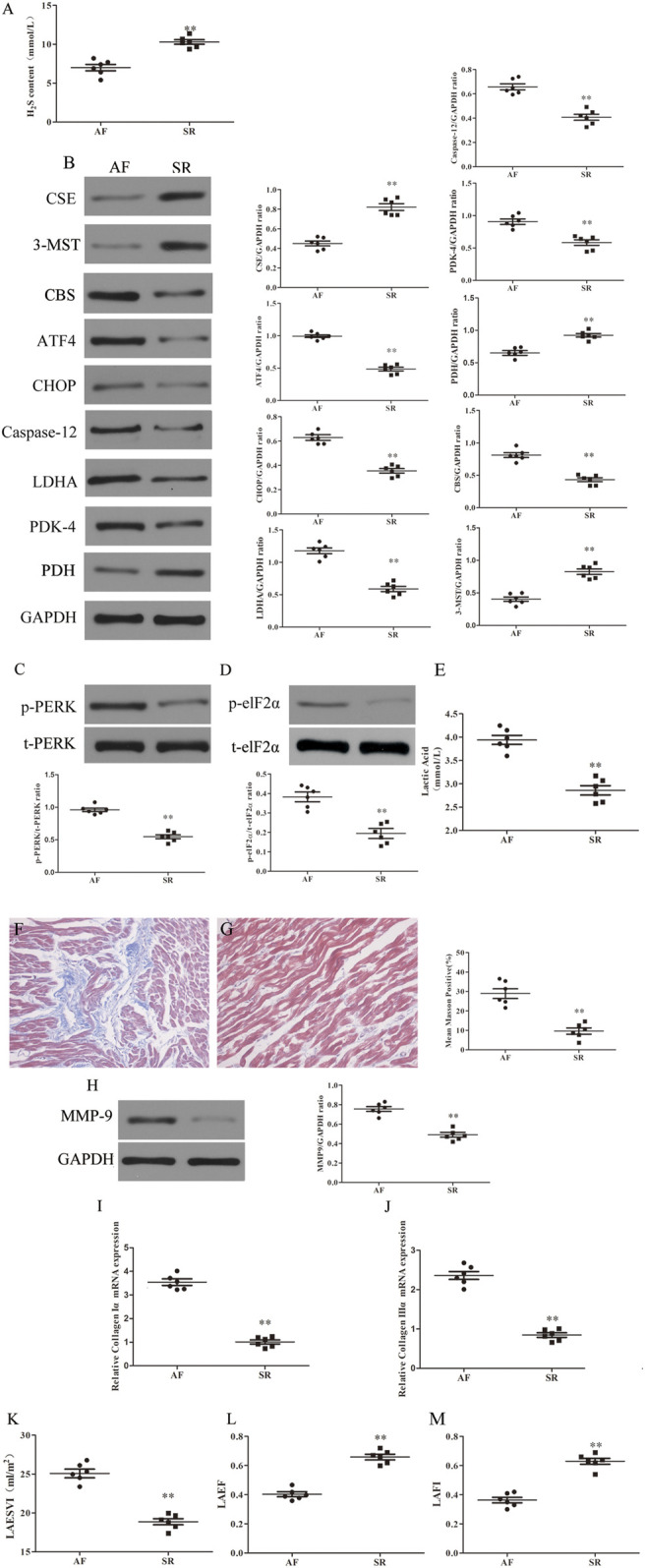
Increased fibrosis, ERS and Warburg effect and decreased CSE expression and left atrial function were found in AF patients compared with SR patients. **(A)** H_2_S content was determined by Methylene blue method in the LAA of patients with SR or AF (*n* = 6 in each group). **(B)** Representative Western blotting and relative densitometry analysis of CSE, CBS, 3-MST, PDK-4, LDHA, PDH, ATF-4, CHOP, caspase-12 and GAPDH in the LAA of patients with SR or AF (*n* = 6 in each group). **(C)** Western blotting was used to analyze the protein levels of p-PERK and t-PERK in the LAA of patients with SR or AF (*n* = 6 in each group). **(D)** Western blotting was used to analyze the protein levels of p-elf2*α* and t-elf2*α* in the LAA of patients with SR or AF (*n* = 6 in each group). **(E)** Lactate acid was determined by kit analysis in the LAA of patients with SR or AF (*n* = 6 in each group). Blue staining with representative Masson staining (scale bar = 50 μm) and quantification of atrial fibrosis (*n* = 6 in each group, with ≥40 fields in each group) were used in the **(F)** AF group and **(G)** SR group. **(H)** Western blotting was used to analyze the protein levels of MMP-9 and GAPDH in the LAA of patients with SR or AF (*n* = 6 in each group). Representative RT-PCR and relative densitometry analysis of **(I)** collagen I*α* and **(J)** collagen III*α* in the LAA of patients with SR or AF (*n* = 6 in each group). Left atrial function was measured by color Doppler echocardiography **(K)** LAESVI, **(L)** LAEF and **(M)** LAFI (*n* = 6 in each group) in the patients with SR or AF during the echocardiographic studies. **p < 0.01 VS AF. A Student’s t-test was used for each of these comparisons between AF and SR groups.

H_2_S deficiency often leads to the occurrence of a variety of CHDs (Wang et al., 2009). To investigate the role of H_2_S in the development of AF, first we examined the levels of H_2_S in the plasma of RHD patients with AF or SR. We found that plasma levels of H_2_S was significantly lower in patients with AF compared with SR patients (Figure 1A). Second, we detected the expression of H_2_S synthase CSE, CBS and 3-MST in LAA obtained from RHD patients with AF or SR. We found that CSE and 3-MST expression were downregulated and CBS expression was upregulated in the LAA from AF patients compared with SR patients (Figure 1B). In addition, the expression levels of ERS axes, including caspase-12, CHOP, ATF4 (Figure 1B), p-elF2α and p-PERK (Figures 1C,D), and the Warburg effect markers, including LDHA, PDK-4 (Figure 1B) and lactic acid (Figure 1E), were increased in the LAA from AF patients compared with SR patients. Finally, we examined the extent of fibrosis in LAA from patients with AF or SR and we found masson positivity (Figures 1F,G) and the expression of MMP-9 (Figure 1H), collagen Iα and collagen IIIα (Figures 1I,J) were upregulated and cardiac colour Doppler ultrasound indicated that LAESVI was increased (Figure 1J) and LAEF and LAFI were decreased (Figures 1K,L) in AF patients compared with SR patients.

### NaHS Inhibits the Ang II-Induced Atrial Warburg Effect in SD Rats

**FIGURE 2:**
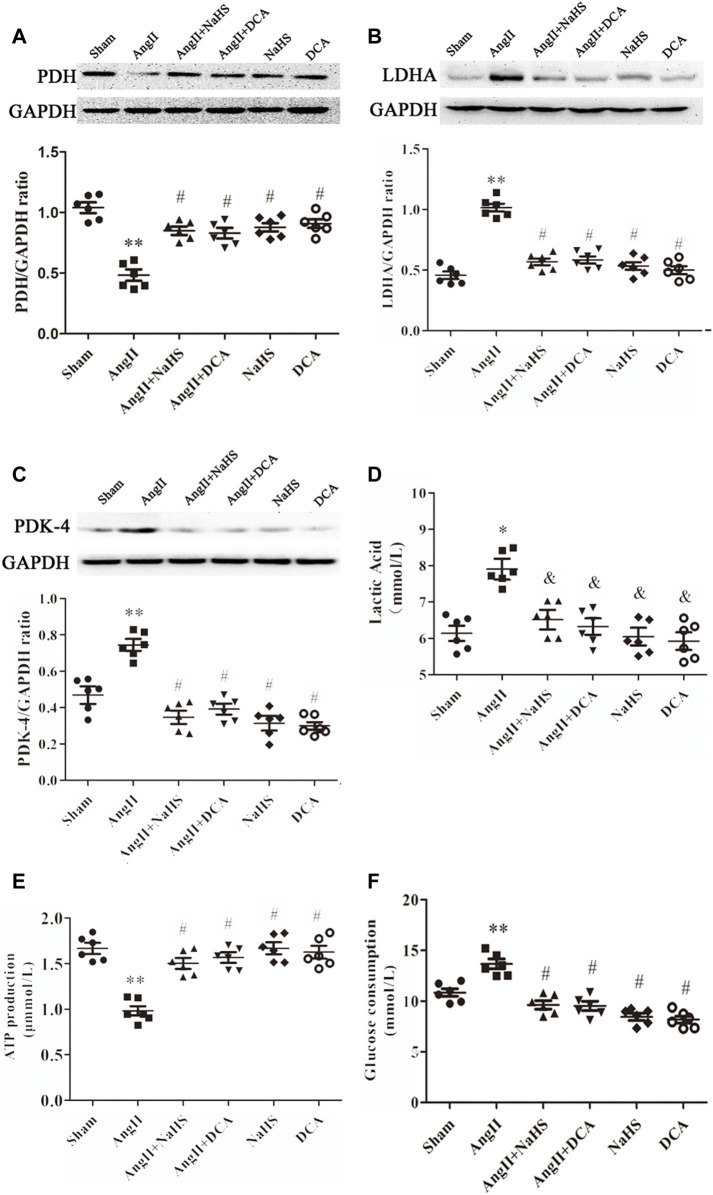
NaHS attenuated the Warburg effect in the left atrium gained from rats under Ang-II treatment. Representative Western blotting and relative densitometry analysis of **(A)** PDH, **(B)** LDHA and **(C)** PDK-4 with GAPDH as a loading control in the rats left atrial tissue of the indicated groups. Lactate acid **(D)** and the production of ATP **(E**) and glucose consumption **(F)** were determined by kit analysis in the rats left atrial tissue of the indicated groups **(** *n* = 6 in each group**)**. **p < 0.01 VS Sham, *p < 0.05 VS Sham, #p < 0.01 VS Ang II, &p < 0.05 VS Ang II. Groups were compared using one-way analysis of variance (ANOVA) (Tukey’s) in **(A–F)**.

Continuous stimulation with Ang-II can lead to the formation of atrial fibrosis (Ge et al., 2020). As shown in Figure 2, the decreased expression of PDH (Figure 2A) and increased expression of LDHA and PDK-4 (Figures 2B,C), accompanied by enhanced local lactate acid accumulation (Figure 2D), decreased ATP production (Figure 2E) and promoted glycogen consumption (Figure 2F) in the left atrium of SD rats with Ang-II infusion, it suggests that the Warburg effect in the left atrium of SD rats is enhanced after Ang-II infusion. Additionally, NaHS supplementation dramatically enhanced PDH expression (Figure 2A) and inhibited the expression of PDK-4 and LDHA (Figures 2B,C), accompanied by decreased local lactate acid (Figure 2D), increased ATP production (Figure 2E) and reduced glycogen consumption (Figure 2F) in the left atrium of SD rats with Ang-II infusion, suggesting that exogenous supplementation with H_2_S was able to prevent the Warburg effect in the left atrium of SD rats after Ang-II infusion. This effect of H_2_S is consistent with the effect of exogenous supplementation with DCA, a specific inhibitor of the Warburg effect key enzyme PDK.

### NaHS Inhibits Ang-II-Induced Atrial ERS in SD Rats

**FIGURE 3:**
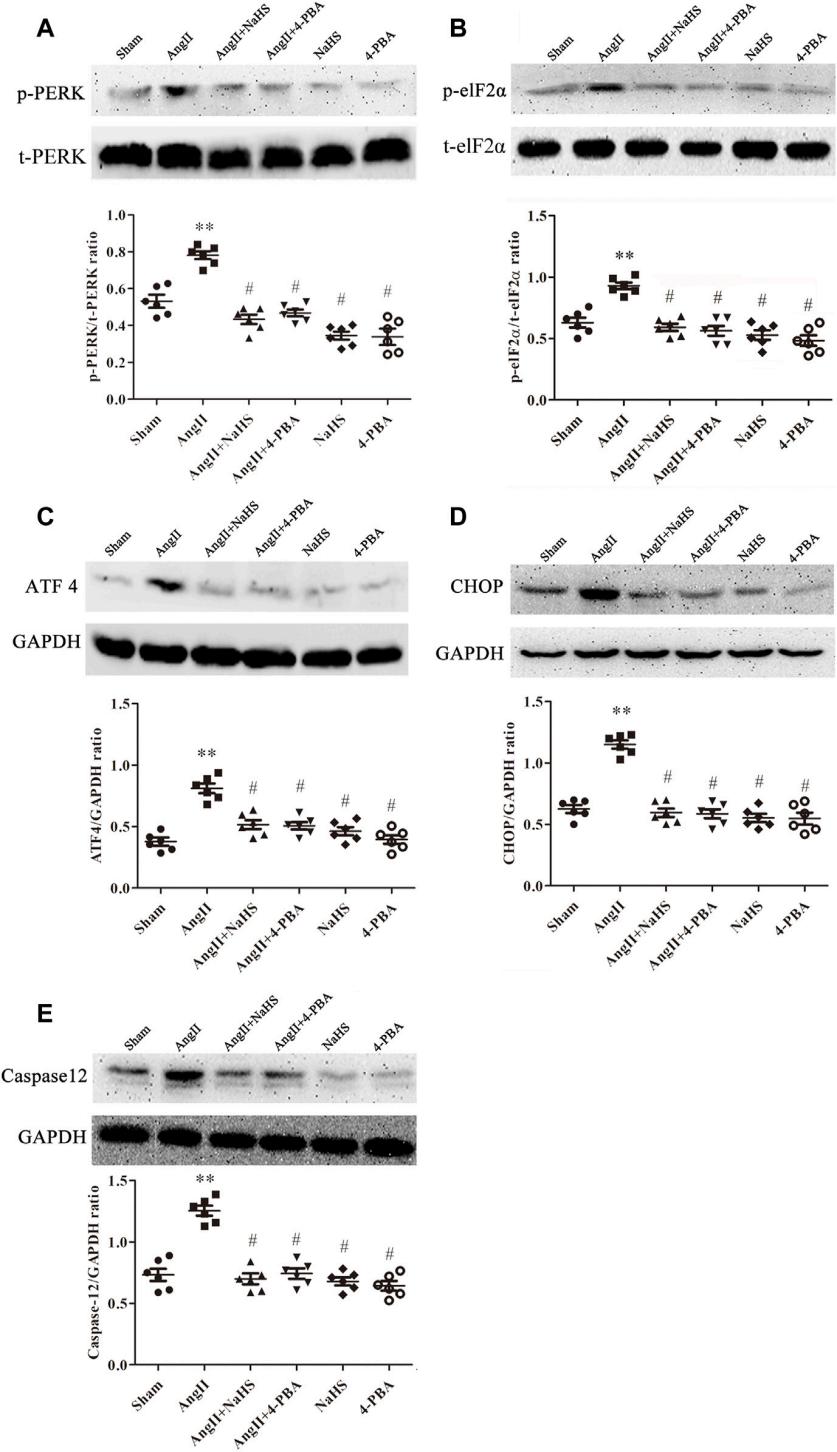
NaHS attenuated the ERS in the left atrium gained from rats under Ang II treatment. Representative Western blotting and relative densitometry analysis of **(A)** p-PERK with t-PERK as the loading control, **(B)** p-elF2*α* with t-elF2*α* as the loading control and **(C)** ATF 4, **(D)** CHOP and **(E)** caspase12 with GAPDH as the loading control in the rats left atrial tissue of the indicated groups. (*n* = 6 in each group) **p < 0.01 VS Sham, #p < 0.01 VS Ang II. Groups were compared using one-way analysis of variance (ANOVA) (Tukey’s) in **(A–E)**.

Apoptosis of cardiomyocytes results in myocardial fibrosis (Zhang et al., 2019). ERS is positively associated with myocardial apoptosis and contributes to the development of myocardial fibrosis, which leads to various CHDs. To determine whether H_2_S prevents atrial fibrosis through inhibition of apoptosis caused by ERS, we treated SD rats upon Ang II infusion with NaHS. As shown in Figure 3, Ang-II induced ERS in the left atrium of SD rats, evidenced by increased expression of p-PERK (Figure 3A), p-elF2a (Figure 3B), ATF4 (Figure 3C), CHOP (Figure 3D) and caspase 12 (Figure 3F). Cotreatment with NaHS significantly inhibited the expression of p-PERK (Figure 3A), p-elF2a (Figure 3B), ATF4 (Figure 3C), CHOP (Figure 3D) and caspase12 (Figure 3F) in the left atrium of SD rats induced by Ang-II infusion, which was consistent with the effect of supplementation with 4-PBA, a specific inhibitor of ERS.

### NaHS Inhibits Left Atrial Remodelling Induced by Ang-II in SD Rats and is Associated With Inhibition of the Warburg Effect and ERS

**FIGURE 4:**
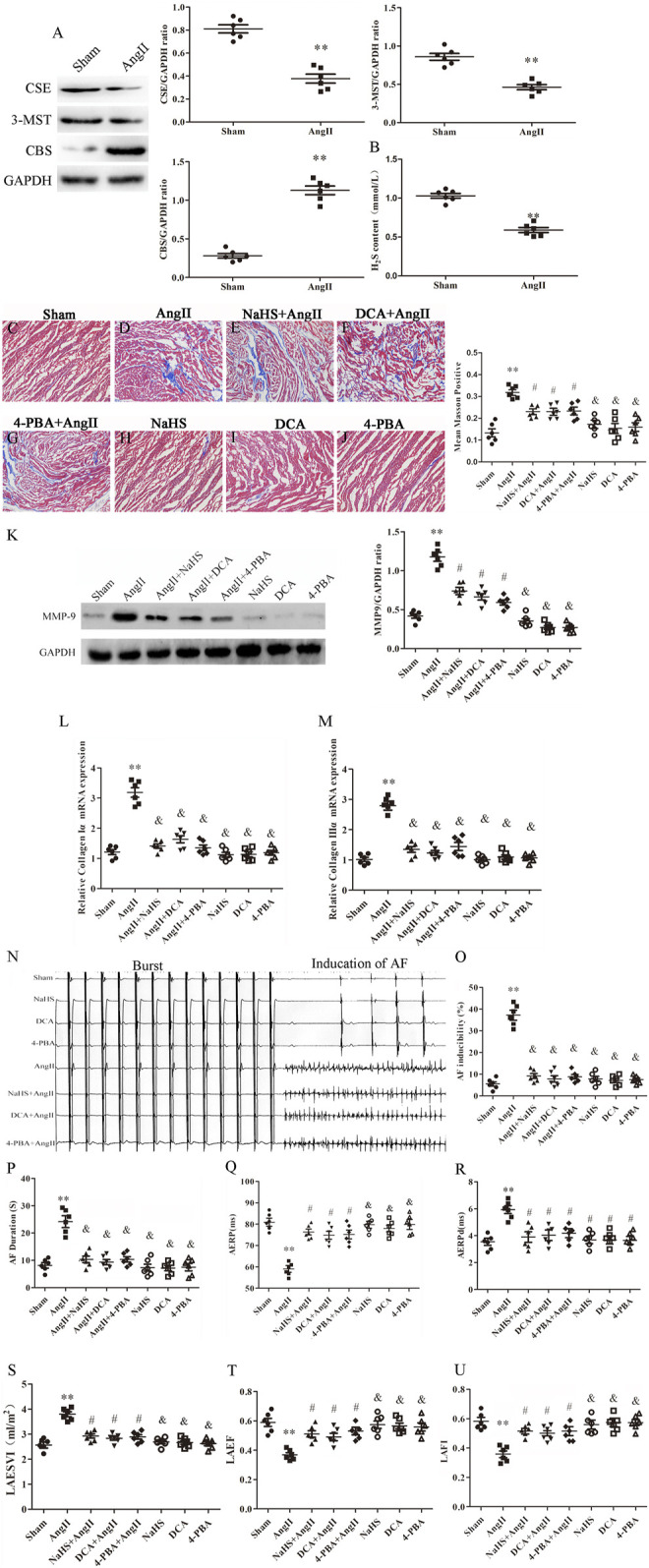
NaHS inhibits left atrial remodeling induced by Ang II in SD rats and is associated with the inhibition of the Warburg effect and ERS. **(A)** Representative Western blotting and relative densitometry analysis of CSE, 3-MST and CBS with GAPDH as the loading control in the rats left atrial tissue of the indicated groups. (*n* = 6 in each group). **(B)** H_2_S content was determined by Methylene blue method in the rats plasma of the indicated groups (*n* = 6 in each group). Blue staining with representative Masson staining (scale bar = 50 μm) and quantification of atrial fibrosis (*n* = 6 in each group, with ≥40 fields in each group) were used in the **(C)** Sham group, **(D)** Ang II group, **(E)** NaHS + Ang II group, **(F)** DCA + Ang II group, **(G)** 4-PBA + Ang II group, **(H)** NaHS group, **(I)** DCA group and **(J)** 4-PBA group. **(K)** Representative Western blotting and relative densitometry analysis of MMP-9 with GAPDH as the loading control in the rats left atrial tissue of the indicated groups (*n* = 6 in each group). Representative RT-PCR and relative densitometry analysis of **(L)** Collagen I*α* and **(M)** Collagen IIIαin the rats left atrial tissue of the indicated groups (*n* = 6 in each group). **(N)** ARPS was performed via the esophagus, and AF episodes and duration were recorded in the rats indicated groups. Electrophysiological analysis of numbers of AF episodes **(O)** and durations of AF **(P)**, AERP **(Q)** and AERPd **(R)** in the rats indicated groups during the electrophysiological studies (*n* = 6 in each group). Left atrial function was measured by color Doppler echocardiography **(S)** LAESVI, **(T)** LAEF, and **(U)** LAFI in the rats indicated groups during the echocardiographic studies (*n* = 6 in each group). **p < 0.01 VS Control, and p < 0.01 VS Ang II, #p < 0.05 VS Ang II. Groups were compared using one-way analysis of variance (ANOVA) (Tukey’s) in C to M and P to U, Fisher’s exact test was used to compare groups in O, and Student’s t-test was used to compare groups in **(A,B)**.

Left atrial remodelling induced by AF involves atrial structural and electrophysiological remodelling (Wang et al., 2019). Myocardial fibrosis causes structural remodelling that can lead to cardiac dysfunction, whereas atrial fibrosis leads to LADS. Lower plasma H_2_S content is closely related to atrial fibrosis. First, we observed that Ang-II infusion significantly decreased CSE and 3-MST expression and increased CBS expression in the left atrium of SD rats (Figure 4A), and decreased H_2_S content in the plasma of SD rats (Figure 4B). In addition, we observed that Ang-II infusion significantly increased atrial fibrosis in SD rats with positive Masson staining, increased MMP-9, collagen Iα and collagen III α expression (Figures 4C–M), and increased susceptibility to AF, including promoted the number of AF episodes and extended AF duration, shortened AERP and prolonged AERPd (Figures 4N–R), and cardiac color Doppler ultrasound confirmed left atrial dysfunction (LADS), including enlarged LAESVI and decreased LAEF and LAFI (Figures 4S–U) compared with saline injected SD rats. Finally, SD rats treated with NaHS, DCA or 4-PBA were protected against Ang II-induced atrial fibrosis, evidenced by negative Masson staining, decreased MMP-9, collagen Iα and collagen III *α* expression (Figures 4C–M), and decreased susceptibility to AF, including reduced number of AF episodes and shortened AF duration, prolonged AERP and shortened AERPd, (Figures 4N–R), and improved LADS, including narrowed LAESVI and increased LAEF and LAFI(Figures 4S–U) compared with Ang-II injected SD rats. However, treatment with DCA or NAHS or 4-PBA alone had no effect on the formation of atrial fibrosis, left atrial function and susceptibility to AF.

### NaHS Inhibited HL-1 Apoptosis Induced by Ang II *via* Prevention of the Warburg Effect and ERS

**FIGURE 5:**
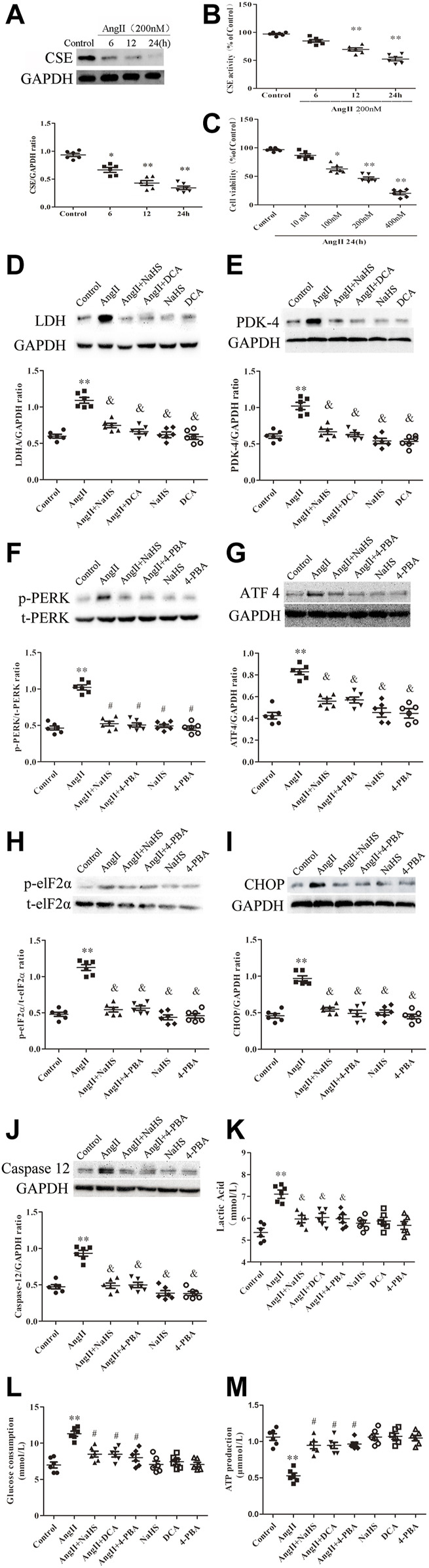
NaHS attenuated the Warburg effect and ERS in HL-1 cells under Ang II treatment. Representative Western blotting and relative densitometry analysis of **(A)** CSE with GAPDH as a loading control induced by Ang-II concentration in the HL-1 cell of the indicated groups (*n* = 6 in each group). **(B)** Detection of CSE activity by methylene blue assay in the HL-1 cell of the indicated groups (*n* = 6 in each group). CCK-8 was used to detect the changes in HL-1 cell viability induced by Ang II at different times **(C)** (*n* = 6 in each group). Representative Western blotting and relative densitometry analysis of **(F)** p-PERK with t-PERK as the loading control and **(H)** p-elF2*α* with t-elF2*α* as the loading control and **(D)** LDH, **(E)** PDK-4, **(G)** ATF 4, **(I)** CHOP and **(J)** caspase12 with GAPDH as the loading control in the HL-1 cell of the indicated groups (*n* = 6 in each group). Lactate acid **(K)** and glucose consumption **(L)** and the production of ATP **(M)** were determined by kit analysis in the HL-1 cell of the indicated groups (*n* = 6 in each group). **p < 0.01 VS Control, and p < 0.01 VS Ang II. Groups were compared using one-way analysis of variance (ANOVA) (Tukey’s) in **(A–J)**.

**FIGURE 6:**
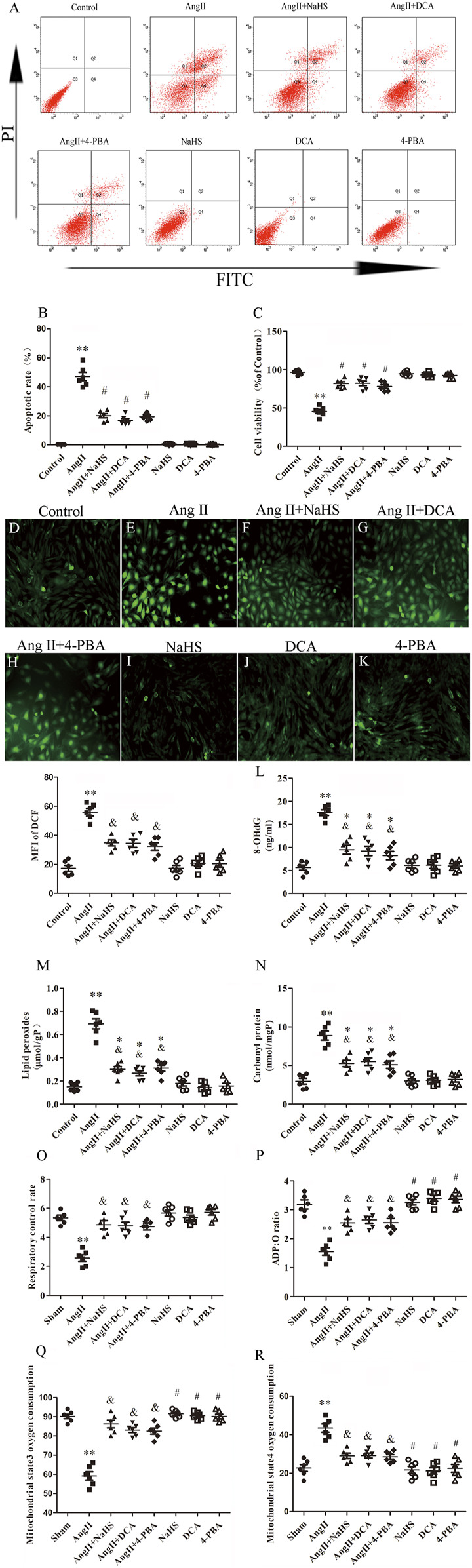
NaHS inhibits HL-1 oxidative stress induced by Ang II and is associated with attenuation of the Warburg effect and ERS. **(A)** Red staining with representative flow cytometry and quantification of the HL-1 cell apoptotic rate **(B)** were used as indication treatments (*n* = 6 in each group). **(C)** CCK-8 was used to detect the changes in HL-1 cell viability induced by the indicated treatments (*n* = 6 in each group). After different treatments, ROS were measured using DCFH-DA staining followed by photofluorography (*n* = 6 in each group, scale bar = 50 μm) and quantitative analysis of the mean fluorescence intensity (MFI) in each group. **(D)** Control, **(E)** Ang II, **(F)** Ang II + NaHS, **(G)** Ang II + DCA, **(H)** Ang II + 4-PBA, **(I)** NaHS, **(J)** DCA and **(K)** 4-PBA. 8-OHdG **(L)**, lipid peroxides **(M)** and Carbonyl protein **(N)** were determined by kit analysis in the HL-1 cell of the indicated groups (*n* = 6 in each group). Mitochondrial oxygen consumption was measured using a Clark-type oxygen electrode to detect **(O)** The respiratory control rate (RCR), **(P)** The ADP/O ratio, **(Q)** The mitochondrial state3 oxygen consumption, **(R)** The mitochondrial state4 oxygen consumption in the HL-1 cell of the indicated groups (*n* = 6 in each group)**p < 0.01 VS Control, *p < 0.05 VS Control, and p < 0.05 VS Ang II, #p < 0.01 VS Ang II. Groups were compared using one-way analysis of variance (ANOVA) (Tukey’s) in **(D–R)**, Fisher’s exact test was used to compare groups in **(B,C)**.

To further determine the roles of H_2_S, ERS and the Warburg effect in HL-1 cell apoptosis, we treated HL-1 cells with Ang-II in the presence or absence of NaHS, DCA or 4-PBA. We found that Ang-II significantly reduced the expression and activity of CSE in a concentration-dependent and time-dependent manner, and decreased cell viability in a time-dependent manner (Figures 5A–C). First, we investigated the expression of key enzymes involved in the Warburg effect and key enzymes involved in ERS, glucose consumption and ATP production, as shown in Figure 5. We found that Ang-II significantly increased the expression of LDH, PDK-4, p-PERK, ATF4, p-elF2α, CHOP and caspase-12 and the lactic acid content in HL-1 cells, and these changes were blocked by treatment with NaHS or DCA or 4-PBA (Figures 5D–K). Treatment with NaHS or DCA or 4-PBA alone did not affect the expression of LDH, PDK-4, p-PERK, ATF4, p-elF2α, CHOP and caspase-12 and the lactic acid content in HL-1 cells (Figures 5D–K). In addition, Ang-II significantly increased glucose consumption and decreased ATP production in HL-1 cells, and these effects were blocked by treatment with NaHS or DCA or 4-PBA (Figures 5L,M). Treatment of HL-1 cells with NaHS, DCA or 4-PBA alone did not affect glucose consumption or ATP productio*n* (Figures 5L,M). Second, using flow cytometry and CCK-8 to detect apoptosis, we further demonstrated that Ang-II significantly promoted HL-1 cell apoptosis and reduced the viability of HL-1 cells, whereas NaHS, DCA and 4-PBA significantly prevented HL-1 cell apoptosis induced by Ang II. Treatment with NaHS, DCA or 4-PBA alone did not affect HL-1 cell apoptosis (Figures 6A–C). Oxidative stress induced ERS in cardiomyocytes. In the present study, we demonstrated that Ang-II significantly increased ROS generation, carbonyl protein expression, 8-OHdG content and lipid peroxides in HL-1 cells, whereas NaHS, DCA and 4-PBA significantly blocked Ang-II-induced ROS production, carbonyl protein expression, 8-OHdG content and lipid peroxides in HL-1 cells. NaHS, DCA and 4-PBA alone did not affect ROS production, carbonyl protein expression, 8-OHdG content or lipid peroxides in HL-1 cells (Figures 6D–N). When mitochondria are damaged by oxidative stress, the production of ATP is reduced. To detect whether mitochondrial function was damaged, we tested mitochondrial respiratory function, including state three and four respiration, RCR and the ADP/O ratio. We observed that the state 3, RCR and ADP/O ratios in Ang II-treated HL-1 cells were significantly reduced compared with those in control HL-1 cells, and state four was significantly increased (Figures 6O–R). Compared with HL-1 cells in the Ang-II group, NaHS or DCA or 4-PBA treatment of HL-1 cells significantly increased RCR, ADP/O ratio and the state 3 (Figure 6Q) and significantly reduced the state 4 (Figure 6R). HL-1 cells treated with NaHS or DCA or 4-PBA alone showed no significant differences in state three or state four or RCR or the ADP/O ratio compared to control HL-1 cells (Figures 6O–R)*.*

## Discussion

AF is a common cause of cardiovascular death and a major public health problem worldwide. Increasing research has demonstrated that atrial fibrosis is a key step leading to the development and progression of AF and is positively correlated with the Warburg effect and ERS activation (Wiersma et al., 2017; Hu et al., 2019). At the same time, the degree of atrial fibrosis is also positively correlated with atrial structural and electrophysiological remodelling. Although the mechanisms by which H_2_S regulates the occurrence and development of AF have been documented, the effects of H_2_S on atrial fibrosis progression to AF induced by the Warburg effect and ERS are still unknown. First, our study revealed that AF reduces serum H_2_S content by inhibiting H_2_S synthase and that AF can promote the Warburg effect and increase ERS in atrial tissue, with increased atrial fibrosis progression to AF. Second, compared with the sham group, the Warburg effect and ERS in the atrial tissue of AF rats treated with NaHS were significantly reduced, and atrial fibrosis progression to AF was inhibited. Finally, through *in vitro* cytology experiments, we found that NaHS reduced HL-1 cell apoptosis by inhibiting the Warburg effect and ER stress occurrence in Ang-II treat HL-1 cell, manifested by improved mitochondrial function and energy metabolism impairment as well as reduced ROS generation. Collectively, these data suggest that H_2_S plays a critical role in regulating atrial structural remodelling and electrical remodelling induced by the Warburg effect and ERS in AF. Our findings establish that H_2_S can be used as a potential therapeutic strategy to prevent the development of AF induced by atrial fibrosis.

The heart is characterized by high energy consumption and oxygen supply and is intolerant of hypoxia. The myocardium requires a sufficient energy supply to maintain normal circulation. Mitochondrial dysfunction is one of the main features of AF. Our previous studies showed that AF leads to an increase in the atrial Warburg effect, which is closely related to atrial fibrosis and increased susceptibility to AF (Hu et al., 2019), and mitochondrial dysfunction is one of the main features of AF. Therefore, when AF occurs, the substrate of atrial energy metabolism is converted from fatty acids to glucose, and energy is provided through the Warburg effect, thereby promoting atrial fibrosis and atrial electrical remodelling and reducing the pumping function of the heart. Current research has verified that atrial fibrosis is the basis for structural remodelling and the persistence of AF. The occurrence and development of atrial fibrosis are inseparable from the excessive deposition of extracellular matrix proteins. Therefore, the balance between atrial fibrosis and anti-fibrosis is disrupted, which is reflected in high expression of collagen 1*α*, collagen 3*α* and MMP-9 in pathological results, and the response of the heart pumping function is an increase in LAESVIZ and decrease in LAEF and LAFI (Begg et al., 2017; Njoku et al., 2018). In addition, Ang II can induce left ventricular insufficiency caused by myocardial infarction or continuous progression of HF, as well as left atrial fibrosis progression to AF (Suo et al., 2019). In the current study, Western blot analysis showed that the expression of PDK-4, LDHA and MMP-9 was higher in AF patients than in SR patients. Masson staining showed that the degree of atrial fibrosis in AF patients was significantly higher than that in SR patients. In addition, collagen 1*α* and collagen 3*α* expression and lactic acid content in the atrial tissue of AF patients were significantly higher than in SR patients, and the left atrial function indices LAESVI, LAEF and LAEI were significantly worse in AF patients than in SR patients. Combined with our previous research reports (Hu et al., 2019), the Warburg effect is related to atrial fibrosis progression to AF and can be used as a target for AF prevention and treatment.

The renin-angiotensin-aldosterone system induces the occurrence of atrial fibrosis by secreting endogenous Ang-II and is believed to play an important role in the occurrence and development of AF (Fan et al., 2015). In animal models, continuous subcutaneous stimulation with Ang II can induce atrial fibrosis in rats and promote the occurrence and development of AF (Ge et al., 2020). In addition, Ang-II induces atrial cell apoptosis, which is the basis of atrial fibrosis (Zhu et al., 2021). In addition to atrial myocytes, the heart also contains ventricular cardiomyocytes, fibroblasts, smooth muscle cells and endothelial cells. The functions of these cells are also regulated by Ang-II. For example, Ang-II has multiple effects on cardiomyocytes and cardiac fibroblasts and can cause cardiomyocyte hypertrophy, cardiac fibroblast proliferation and cardiac interstitial fibrosis. It is particularly noteworthy that interstitial fibrosis is associated with cardiac wall stiffness after myocardial infarction and HF and decreased electrical coupling of myocardial cells, thereby promoting the occurrence of AF (Zlochiver et al., 2008). In addition, endothelial cells are the most numerous cell type among cardiac mesenchymal cells and play an important role in heart remodelling. The phenotypic transition from endothelial cells to mesenchymal cells is called endothelial-to-mesenchymal transition (EndoMT), and endothelial cells mainly regulate myocardial fibrosis through EndoMT. Current studies have confirmed that Ang-II can induce excessive activation of EndoMT in endothelial cells and cause myocardial fibrosis and extracellular matrix protein accumulation (Piera-Velazquez et al., 2011; Zou et al., 2021). In our study, Masson staining showed that the atrial fibrosis in the rats in the Ang-II treatment group was significantly more serious than that in rats in the sham group, and the expression of PDK-4, LDHA, collagen I*α*, collagen III*α* and MMP-9 was significantly increased, the susceptibility to AF was increased, and the left atrial function deteriorated. In the *in vitro* cell experiment, compared with the control group, the HL-1 cell activity in the Ang-II treatment group was significantly decreased, and the degree of apoptosis was significantly increased, accompanied by the expression of PDK-4 and LDHA. Whether *in vivo* or *in vitro*, the use of DCA, a specific inhibitor of the Warburg effect, significantly improved the pathophysiological changes caused by Ang-II treatment. Notably, Scientific studies have identified DCA not only reverses metabolic derangements but also effectively targets mitochondrial related pathways of apoptosis, and significantly improve cardiomyocyte apoptosis and myocardial fibrosis in rats with right ventricular cardiomyopathy (Sun et al., 2016). Our previous research results show that DCA alone will not increase atrial fibrosis in normal dogs (Hu et al., 2019). Then, we further explored the relationship between the Warburg effect and atrial fibrosis caused by atrial cell apoptosis.

Disruption of ER homeostasis triggers ERS, which can be restored by activating the UPR in the short term. Activation of CHOP by the upstream PERK-eIF2*α*-ATF4 axis plays an important role in ERS-related apoptosis in cardiomyocytes (Fels and Koumenis, 2006). CHOP activates a series of signal transduction pathways to upregulate the expression of caspase-3, ultimately leading to apoptosis (Feng et al., 2019). When the myocardium develops lesions, the oxidation of beta fatty acids is limited, and instead these fatty acids provide energy for glucose metabolism. In the myocardium of diabetic rats, disturbed glucose metabolism leads to downregulation of the expression of glycometabolic proteins, such as PPARα and PGC-1*α*, which in turn activate the expression of p-PERK, p-eIF2*α*, ATF6, CHOP and ATF4, suggesting that disturbed glucose metabolism is a key step in the activation of myocardial ERS (Lakshmanan et al., 2013). In the AF state, myocardial tissue obtains energy through the Warburg effect, leading to tissue microenvironment acidosis. On the one hand, acidosis can directly reduce the activity of myocardial ER and further aggravate the occurrence of ERS and cardiac fibrosis and remodelling (Glembotski et al., 2012; Yong et al., 2019); on the other hand, acidosis can induce ERS in many cell types; for example, acidosis can lead to ERS and induce cardiac fibrosis in patients with HF (Schaffer and Kim, 2018). It should be noted that ERS is involved in the occurrence of AF (Wiersma et al., 2017). In our study, Western blotting analysis revealed that the expression of the p-PERK-p-eIF2-*α*-ATF4-CHOP-Caspase 12 axis in the left atrial tissue of patients with atrial fibrillation was significantly increased compared with that in SR patients and that Ang-II treatment significantly increased the expression of the p-PERK-p-eIF2-*α*-ATF4-CHOP-Caspase 12 axis in rat left atrium tissue and HL-1 cells. The ERS-specific inhibitor 4-PBA inhibited the Ang-II-induced p-PERK-p-eIF2-*α*-ATF4-CHOP-Caspase 12-axis expression in HL-1 cells and rat left atrial tissue, improving atrial fibrosis, AF susceptibility and deterioration of left atrial function and reducing HL-1 cell apoptosis. 4-PBA has been approved by the U.S. Food and Drug Administration (FDA) as an ammonia scavenger for children with urea cycle disorders. It is a low molecular weight fatty acid and non-toxic pharmacological compound, and has been proved to promote myocardial repair and prevent myocardial fibrosis after MI (Luo et al., 2015). Therefore, the use of 4-PBA alone will not lead to myocardial fibrosis.

Alterations in mitochondrial energy metabolism contribute to the development of cardiac fibrosis-related diseases (Mihm et al., 2007). Glucose, the other major energy substrate of the heart, is oxidized via glycolysis and converted into energy in the mitochondria. Our previous study showed mitochondrial tricarboxylic acid cycle dysfunction in the development of AF, and such changes resulted in increased myocardial O2 consumption and reduced cardiac efficiency (Yamada et al., 2006). In addition, mitochondria are the main production site of ROS, and mitochondrial proteins, lipids, and mtDNA are considered the main targets of oxidative damage after excessive release of mitochondrial ROS. Moreover, when mitochondrial ROS are abundantly produced, they can induce a large release of Ca^2+^ into the cytoplasm, causing the occurrence of ER stress and promoting mitochondrial membrane permeability transition pore opening, which allows cytoplasmic Ca^2+^ to enter the mitochondria and further accumulate, creating a vicious cycle (Harisseh et al., 2019). The current study revealed that Ang-II increased mitochondrial oxidative stress and mtDNA damage in cardiomyocytes and interstitial cells, leading to cardiac fibrogenesis, cardiac hypertrophy, and apoptosis (Meng et al., 2018). In this study, we also confirmed that Ang-II induced the expression of reactive oxygen species, carbonyl proteins, 8-OHdG, and lipid peroxides; promoted a significant increase in cardiomyocyte apoptosis; and impaired mitochondrial respiratory function and energy metabolism in HL-1 cells. This suggests that both the Warburg effect and ER stress ultimately occur in response to mitochondrial oxidative stress that causes cardiomyocyte injury and thus effectively protect cardiomyocytes from oxidative damage, which is the direction of our further in-depth research.

Endogenous H_2_S is the final metabolite produced by H_2_S synthase in the body to convert sulfur-containing amino acids. It exists in gaseous form and as dissolved NaHS. NaHS can be hydrolysed into Na^+^ and HS^−^ in the body, the latter of which can produce H_2_S. The current research on the role of H_2_S in animal models uses the exogenous H_2_S donor NaHS, which can be used to increase the level of H_2_S in plasma and the content of H_2_S in the myocardium. In recent years, it has been generally believed that H_2_S exerts cardioprotective effects through antiapoptotic, anti-inflammatory and antifibrotic properties, especially in CHD, HF, AF and other diseases characterized by myocardial fibrosis. Our current research combined with our previous research reports (Hu et al., 2019) found that both Warburg effect and ERS can lead to atrial myocyte apoptosis, which is closely related to the inflammatory damage caused by oxidative stress. Therefore, this is one of the main ways to explore whether H_2_S can improve the occurrence of AF. First, we compared the difference between H_2_S synthase and H_2_S content in patients with AF and SR. Studies have shown that when AF occurs, stable sulfides stored in the mitochondria are mobilized to generate free sulfides in the blood and used to counteract myocardial damage caused by oxidative stress (Watts et al., 2021). Therefore, the longer the AF occurs, the more the long-term stable sulfide storage reserve is exhausted, resulting in a decrease in the H_2_S content in the plasma and an intensified oxidative stress response, thereby aggravating the occurrence of atrial fibrosis (Watts et al., 2021). Our study also confirmed lower CSE expression and significantly higher atrial fibrosis in AF atrial tissue than in SR atrial tissue. Interestingly, the CBS levels in AF patients were significantly higher than those in SR patients, which may be related to CSE and CBS feedback regulation (Nandi and Mishra, 2017). We often think that CBS is mainly expressed in the nervous system, but current studies have confirmed that this enzyme also exists in the cardiovascular system and exerts a cardiovascular protective effect (Bucci et al., 2014). When glucose metabolism is abnormal, CBS is upregulated in myocardial tissue and myocardial interstitial tissue. Unfortunately, this increase in CBS is not sufficient to cause an increase in overall H_2_S production. In contrast, because the expression of CSE and 3-MST is reduced, the overall H_2_S content is still low; thus, myocardial fibrosis is still developing at this time (Jin et al., 2015). In the SD rat model, we also further confirmed that SD rats treated with Ang-II can significantly reduce the expression of CSE and 3-MST, increase the expression of CBS, and increase the occurrence of atrial fibrosis. Regrettably, although the expression of CBS increased, the content of H_2_S in the plasma of SD rats induced by Ang-II did not increase significantly, but decreased, which is consistent with the study of Watts (Watts et al., 2021). It is suggested that CSE is the main synthase of H_2_S in the cardiovascular system, and the decrease of H_2_S content induced by Ang-II is closely related to atrial fibrosis.

Previous studies have reported that db/db mice are susceptible to diabetic cardiomyopathy, which is related to the decrease of H_2_S content in the circulation and glucose metabolism disorders (Sun et al., 2018). H_2_S can improve mitochondrial function and glycolysis and promote aerobic oxidation of glucose in mitochondria to provide energy. It is worth noting that when mitochondrial dysfunction occurs, the tricarboxylic acid cycle is inhibited, thereby promoting the remodelling of myocardial fibrosis, which is manifested by upregulation of MMP-9 and collagen I expression (Liang et al., 2015). At this time, tricarboxylic acid cycle disorder can also activate autophagy-related proteins and ERS to promote myocardial fibrosis, and this pathophysiological effect of mitochondrial dysfunction can be reversed by NaHS (Kumar et al., 2019; Nie et al., 2021). Ang-II inhibits miR-133a and induces mitochondrial oxidative stress and mtDNA damage in cardiomyocytes, and these Ang-II-induced pathophysiological effects can also be reversed by NaHS (Ceylan-Isik et al., 2013; Su et al., 2021). Our previous research results show that the Warburg effect inhibits the key enzymes of TCA and causes the occurrence of AF (Hu et al., 2019). However, the significantly increases expression of PDK-4, a key Warburg effect enzyme, is the key to this change. PDK-4 has strong activity in glucose metabolism disorders, can activate AF and inhibit the expression of MMP genes, and promote the synthesis of extracellular matrix (Ding et al., 2017). Based on this finding, we found that exogenous H_2_S supplementation can significantly reduce the expression of PDK-4 induced by Ang-II and improve the Warburg effect, which is manifested by reducing the expression of LDHA, reducing the content of lactic acid and abnormal glucose consumption, and increasing the production of ATP. PDK4 is not only a key enzyme of the Warburg effect, but also an important mitochondrial matrix enzyme in cell energy regulation, and is closely related to mitochondrial function. The high expression of PDK-4 leads to the increase of local tissue lactic acid content, which causes the expression of a variety of ERS genes, such as the expression of CHOP, ATF4 and p-eIF2*α* (Dong et al., 2017; Ma et al., 2020). Consistent with those study, our research shows that H_2_S can improve the Warburg effect and mitochondrial function by inhibiting PDK-4 expression, reducing mitochondrial ROS and oxidative stress, and it’s also by inhibiting p-PERK-p-eIF2-*α*-ATF4 -CHOP-Caspase12 axis to reduce the occurrence of ERS and atrial muscle cell apoptosis, thereby improving the progression of atrial fibrosis to AF.

Current research shows that ROS-induced oxidation of ryanodine receptors promotes SR calcium leakage and AF (Xie et al., 2015). Impaired glucose metabolism also leads to mitochondrial oxidative stress, which causes increased MMP-9 expression to promote impaired left atrial function and cause AF (Liang et al., 2018; Gong et al., 2020). In the atria of CSE-KO mice, the level of superoxide was found to increase, which was negatively correlated with changes in H_2_S content and positively correlated with atrial action potential and AF inducibility (Watts et al., 2021). In addition, ROS can cause atrial electrical remodelling by activating the ultrafast outward rectifying potassium current (Ikur), thereby causing AF (Christophersen et al., 2013). It is worth noting that even in myocardium with impaired glucose metabolism, oxidative stress and related structural remodelling are the basis of abnormal electrical activity. H_2_S improves mitochondrial oxidative stress, respiratory dysfunction, and reduces apoptosis in HL-1 cells caused by glucose metabolism disorder, and in turn can improve atrial function in mice (Garcia et al., 2017; Lee et al., 2019). This has similar effects to reducing oxidative stress to inhibit HL-1 cell apoptosis and improve left atrial structural remodelling induced by AF in mice (Johnston et al., 1990). Moreover, studies have confirmed that H_2_S can reduce the instantaneous outward potassium current, maintain the stability of cardiac electrical activity, and shorten the action potential of rat atrial myocytes caused by H_2_S deficiency but does not affect normal atrial electrical activity, so as to improve the occurrence of atrial fibrillation. This phenomenon has also been proved in the mouse atrial fibrillation model (Sheng et al., 2013; Xue et al., 2020; Watts et al., 2021). In our study, the reduction in H_2_S levels was related to the Warburg effect in the atrium and the activation of endoplasmic reticulum stress to induce oxidative stress and led to an increase in susceptibility to AF. Supplementation with H_2_S can significantly reduce oxidative stress and improve susceptibility to AF.

In summary, H_2_S has been shown to improve left atrial dysfunction and AF susceptibility by inhibiting AngII-induced Warburg effect and ERS-induced atrial fibrosis, thereby reducing the occurrence and development of AF. Our research also provides a new strategy involving the use of H_2_S synthase as a key factor in the diagnosis and treatment of atrial fibrosis-related AF and as a new therapeutic target for these diseases. First, there may be other signalling pathways that regulate the Warburg effect and ERS-related atrial fibrosis, such as the MAPK pathway. Whether H_2_S regulates the Warburg effect and endoplasmic reticulum stress through signalling pathways affecting atrial fibrosis should be determined in further studies. In addition, we did not conduct high and low expression studies on endogenous H_2_S synthase. Therefore, to obtain more convincing results, a sufficient number of rats with conditional cardiac knockout and high expression should be created in subsequent studies.

## Data Availability

The original contributions presented in the study are included in the article/supplementary material; further inquiries can be directed to the corresponding author.
