# Supplementary figures and images for: Hydrogen Sulfide Ameliorates Angiotensin II-Induced Atrial Fibrosis Progression to Atrial Fibrillation Through Inhibition of the Warburg Effect and Endoplasmic Reticulum Stress
Source: Front Pharmacol. 2021 Dec 7;12:690371. doi: 10.3389/fphar.2021.690371 (PMC8689064; doi:10.3389/fphar.2021.690371)

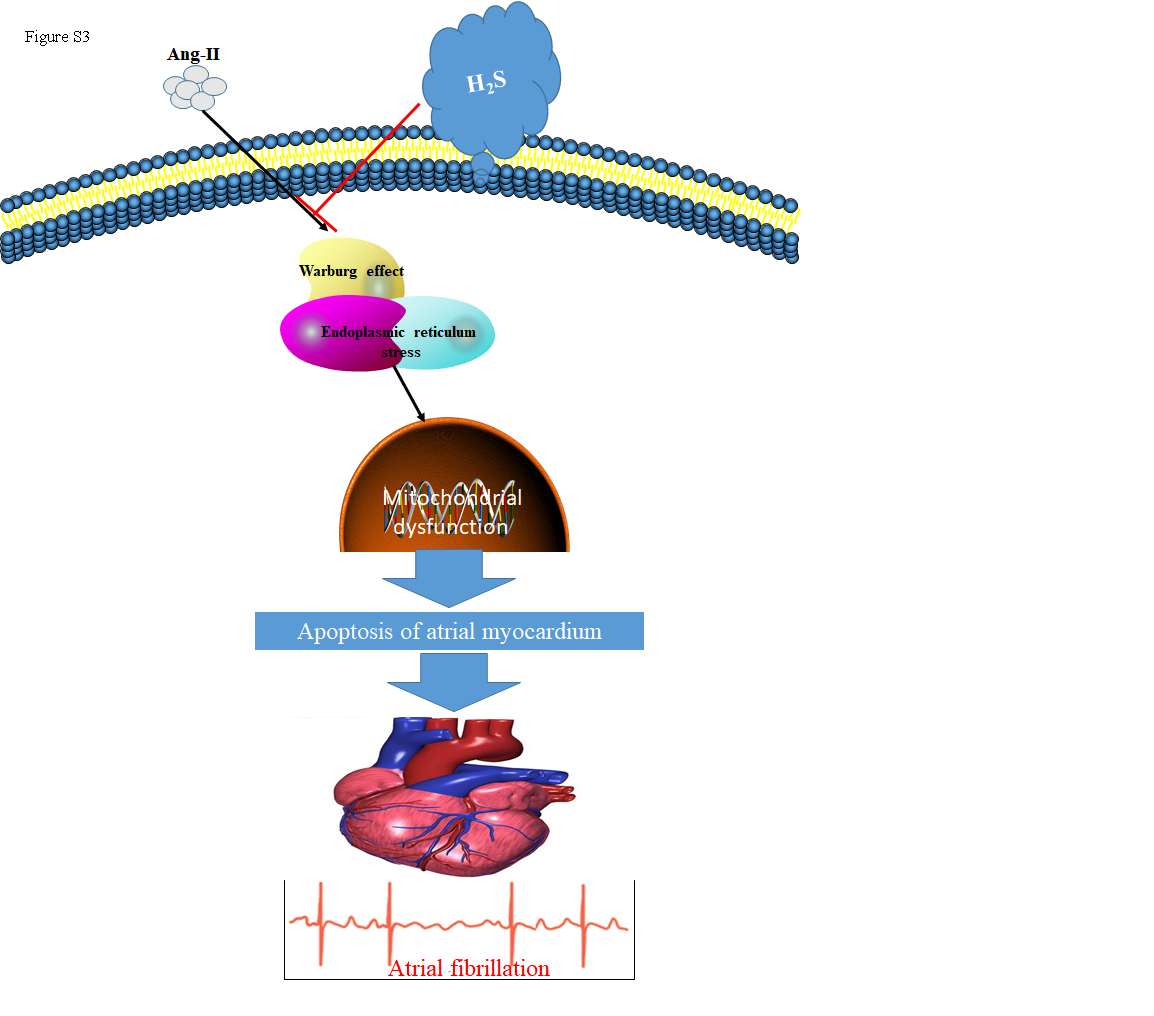

Supplement: Supplementary file 1 [file Image3.TIF]

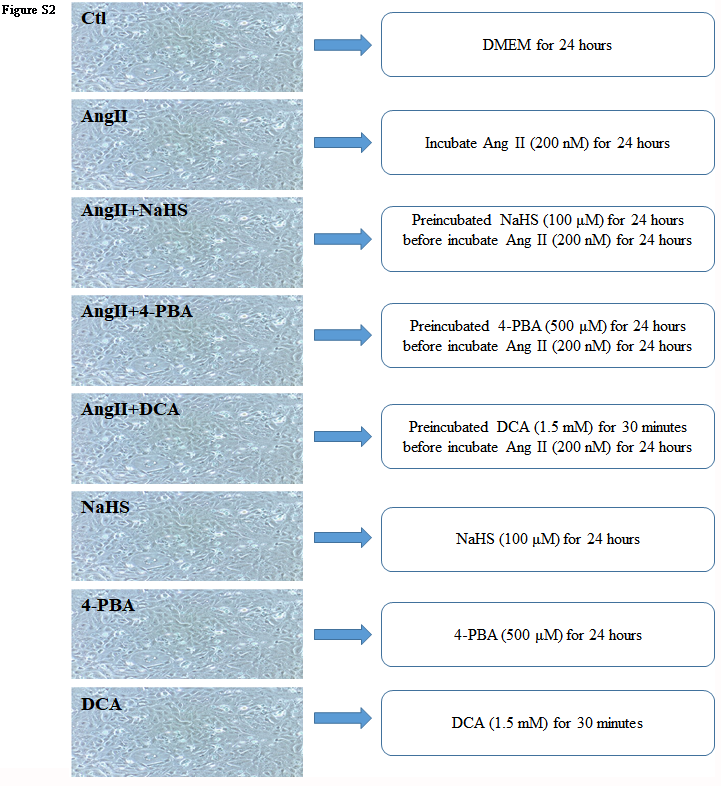

Supplement: Supplementary file 2 [file Image2.TIF]

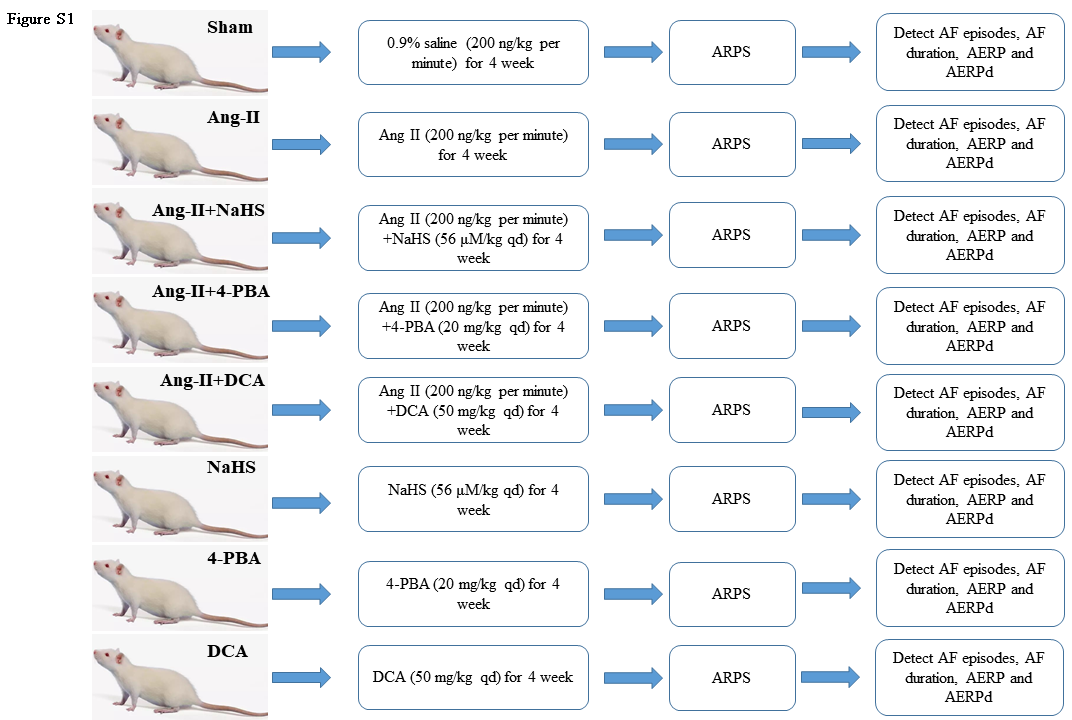

Supplement: Supplementary file 3 [file Image1.TIF]
