# Supplementary material for: Hydrogen Sulfide Ameliorates Angiotensin II-Induced Atrial Fibrosis Progression to Atrial Fibrillation Through Inhibition of the Warburg Effect and Endoplasmic Reticulum Stress
Source: Front Pharmacol. 2021 Dec 7;12:690371. doi: 10.3389/fphar.2021.690371 (PMC8689064; doi:10.3389/fphar.2021.690371)
Supplement: Supplementary file 4 [file DataSheet1.PDF]

Ethics Committee of the First Affiliated Hospital of University of South China

（2018）Ethical review number（S2018016）

1. A total of 12 patients who had mitral valve replacement and underwent coronary angiography to exclude CHD were enrolled according to the following principles:

i. Voluntary principle: the risk and benefit the subject or other persons may suffer as reported by the investigator, and the subject voluntarily participates (signed informed consent).Subjects are entitled to withdraw from the trial at any time during any phase of the trial without discrimination or reprisal and their medical treatment and equity will not be compromised;

ii. Principles of confidentiality: participation in the trial and the availability of personal data in the trial shall be confidential.Only the data of subjects enrolled in the trial can be accessed by the superior administration and ethics committee as specified;

iii. Safety principles: the concern for the benefit of the subjects should be higher than self-scientific and societal considerations, serious adverse events occur in the trial and should be promptly reported to the ethics committee.

iv. Principles of compensation: when trial related damage occurs, subjects can receive treatment and corresponding compensation.

2. 48 SD rats' welfare and experimental procedures were carried out in accordance with the Guide for the Care and Use laboratory Animals (Ministry of Science and Technology, China, 2006)

The above studies passed the ethics committee of the First Affiliated Hospital of the University of South China, and the investigators were asked to carry out the research work strictly in accordance with the approved research protocol, and if there are modifications, the ethics committee should be submitted for discussion and approval.

The Ethics Committee of

The First Affiliated Hospital of the University of South China

2018 年 3 月 1 日
